# Disparities in the quality of and access to services in children with autism spectrum disorders: a structural equation modeling

**DOI:** 10.1186/s13690-021-00577-5

**Published:** 2021-04-26

**Authors:** Mohammad Asghari Jafarabadi, Kamal Gholipour, Hassan Shahrokhi, Ayyoub Malek, Akbar Ghiasi, Hamid Pourasghari, Shabnam Iezadi

**Affiliations:** 1 Department of Statistics and Epidemiology, Faculty of Medicine, Zanjan University of Medical Sciences, Zanjan, Iran; 2 Center for the Development of Interdisciplinary Research in Islamic Sciences, and Health Sciences Tabriz University of Medical Sciences, Tabriz, Iran; 3 Social Determinants of Health Research Center, Tabriz University of Medical Science, Tabriz, Iran; 4 Research Center of Psychiatry and Behavioral Science, Tabriz University of Medical Sciences, Tabriz, Iran; 5 HEB School of Business & Administration, University of the Incarnate Word, San Antonio, TX USA; 6 Hospital Management Research Center, Iran University of Medical Science, Tehran, Iran

**Keywords:** Autism Spectrum disorders, Disparities, Access, Quality, Social determinants of health, Structural equation modeling

## Abstract

**Background:**

Socioeconomic disparities in health and healthcare are global issues that affect both adults as well as children. Children with exceptional healthcare needs, especially those with developmental impairments, including Autism Spectrum Disorders (ASD), encounter major disparities in access to and quality of health services. However, disparities in the population of children are rarely studied. The main aim of this paper is to study the socioeconomic disparities in children with ASD by examining the association between their Social Determinants of Health (SDH) status and access to and the quality of services.

**Methods:**

This is a cross-sectional study on 202 children with ASD conducted in 2019 in two provinces including Ardabil and East-Azerbaijan, in the North-West of Iran. A structured, valid questionnaire was used to collect data on demographic, SDH status, quality of services, and access to services in a population of children with ASD aged 2–16-year-old. Around 77% participants were male and the mean age of children was 2 years and 6 months. Structural Equation Modeling (SEM) were used to assess the relationship.

**Results:**

Based on the results of this study, the overall mean scores of the quality of services, access to services, and SDH status were 61.23 (30.01), 65.91 (21.89), and 29.50 (22.32) out of 100, respectively. All the associations between the quality and access dimensions and quality (B: 0.464–0.704) and access (B: 0.265–0.726) scales were statistically significant (*P* < 0.001). By adjusting to covariates, the access was also significantly related to service quality (*P* = 0.004). Finally, the associations between SDH score with service quality (*P* = 0.039) and access (*P* < 0.001) were positively significant.

**Conclusions:**

There are socioeconomic disparities in the quality of and access to services among children with ASD, who use ASD services, in the North-West of Iran. We recommend health/medical centers, where children are diagnosed with ASD, conducting SDH screening and providing families of low-SDH status with specific information about the quality of and access to services for children with ASD. Additionally, medical universities must have a plan to routinely monitor the quality of and access to services provided for the children with low SDH.

**Supplementary Information:**

The online version contains supplementary material available at 10.1186/s13690-021-00577-5.

## Background

Children with Autism Spectrum Disorders (ASD) experience lifelong neurodevelopmental impairments interfering with their ability to social interactions and communication. Appropriate services and supports could improve the behavioral and cognitive functioning of children with ASD [1], however, one specific service doesn’t fit all children with ASD, and families often express interest in a broad array of interventions when searching for best services for their child with ASD [2].

According to evidence provided by the World Health Organization (WHO), individuals with ASD have higher rates of forgone health-care needs compared to the rest of the population, and their access to services and supports is inadequate [3]. On the other hand, access to healthcare for children with ASD can be affected by race/ethnicity, education level, and the type of insurance [2]. Although there is no evidence to conclude that autism occurrence depends on the families’ characteristics, such as ethnicity, income, lifestyle, and education [4], some studies on the association among sociodemographic characteristics, diagnosis, and health care services for children with ASD have revealed socioeconomic and racial/ethnic disparities [5–7].

Access to evidence-based, high-quality services for children with ASD is a major public health issue [8]. In the previous decades, major efforts were made to improve children’s overall access to health care [9, 10]. Nonetheless, disparities persist in access, utilization, and the quality of care for certain subgroups of children, including those who are Black and Latino, live in lower-income households, and have greater functional limitations [11]. Studies showed that in a population of children with exceptional healthcare needs, children with developmental impairments, including autism, have encountered disparities in health services [12, 13], and, in a population of children with developmental impairments and other exceptional healthcare needs, children with ASD face major challenges to achieve desired services [14, 15]. Lack of access to services and supports is an important issue for people with autism, but in low- and middle-income countries (LMICs) there is a lack of data on this problem [16].

Another important issue in providing services for children with ASD is inequality in the quality of services. Quality of services is associated with patients’ satisfaction with care as well as ease of service use, both could result in better health outcomes [17]. Quality of health care is described as a measure of families’ perceptions of their children’s care and satisfaction with the health services provided according to acceptable cultural norms and values [11, 18]. Disparities in healthcare for children with ASD not only exist in access to services but in the quality of healthcare as well. For example, a study by Montes and Halterman (2011) illustrated that families of children with ASD had less chance to receive family-centered care compared to families without children with ASD. Additionally, disparities were shown between black and white families with children with ASD [18].

Socioeconomic disparities in health and healthcare are global issues that affect both adults as well as children [8]. However, socioeconomic disparities in the population of children are rarely studied [8, 19], especially in LMICs. There is a large gap in evidence; most studies exploring disparities in the quality of or access to healthcare in children with ASD are from developed countries, almost the Unite States (US) [19], and to our knowledge, there is no evidence from Iran to explore such disparities, neither in children with ASD nor those with other lifelong disorders. This issue is particularly important when taking a glance on statistics presented by World Bank in 2017, which shows that more than 61% of all children in the world live in LMICs [20]. A systematic review by Bishop-Fitzpatrick and Kind showed that most studies on disparities have focused on race, and are particularly from the US [19]. Therefore, more researches are needed from LMICs.

In Iran, most special services needed by children with ASD, particularly speech and occupation therapies and family training programs, are not delivered by public centers/clinics. Such services are delivered by independent, private organizations affiliated to welfare organizations. Furthermore, health insurance doesn’t have coverage for ASD rehabilitation services [21]. These issues rise questions on the equity in the quality of and access to services for children with ASD. Do all children with ASD and their families, irrespective of their socioeconomic status, have equal access to services needed for their special conditions? Do they equally receive high-quality services? To explore disparities in the quality of and access to services provided for children with ASD, we will examine the associations between these factors and children’s Social Determinants of Health (SDH).

**Fig. 1:**
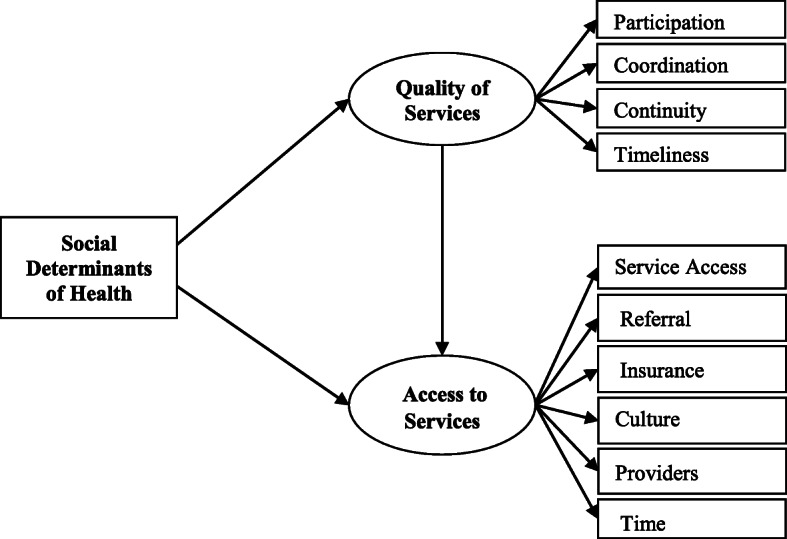
A conceptual model of association between social determinants of health and the quality of and access to services among children with autism spectrum disorders, North-West of Iran, 2019

A conceptual model of SDH /Access/Quality of care was developed to explain and predict the quality of care and access to service based on SDH status of children with ASD [22–24] (Fig. 1), assuming two pathways: 1) predicting the quality of services by SDH pathway and 2) predicting access to services by the quality of services pathway. SDH status of the caregivers explains the perceived quality of services by them. On the other hand, quality of services is a determinant of service utilization and access to care.

## Method

### Design of the study

This project is a part of Azeri Blue Buddies: Interdisciplinary Longitudinal Autism Researches (ABBILAR). This is a cross-sectional study conducted in two provinces, Ardabil and East-Azerbaijan, in the North-West of Iran. We examined associations among SDH status, access to, and the quality of services among children with ASD. Data were collected from July 1 to November 30, 2019, in all educational, treatment, and rehabilitation centers, where children with ASD receive their special services such as rehabilitation and behavioral interventions.

### Sample size calculation

The minimum sample size was estimated 156 participants, given the number of observed variables (10) and latent variables (3) in the model, the anticipated effect size (0.05), and the desired probability (0.05) and statistical power levels (0.95) [25]. We recruited all eligible participants, who were willing to take part in the study, and the sample size increased to 202.

### Participants and sampling

**Fig. 2:**
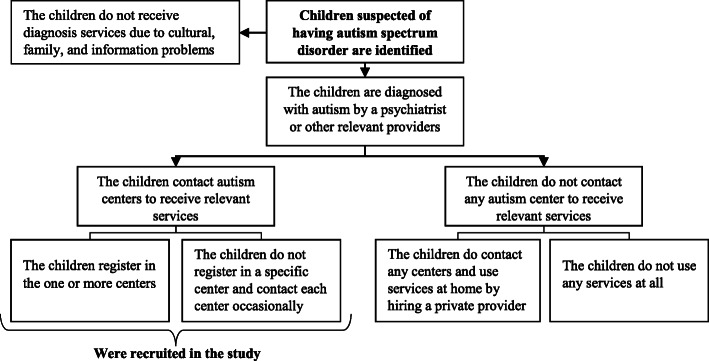
Types of the participants recruited in the study to examine the disparities in the quality of and access to services among the children with autism spectrum disorders, North-West of Iran, 2019

The target population was children with ASD and their families. Children aged 2–16-year-old with an ASD diagnosis document verified by a psychiatrist and who had received at least one visit in the past six months were eligible to participate in the study. We selected the former criterion because centers/clinics provide services for children with ASD aged 2–16-year-old, and, we selected the latter criterion because the participants were supposed to evaluate the quality of and access to services, therefore, they needed to have a minimum level of utilization of services. We used a history of a minimum of six months period to having contact with a center (and no longer) to avoid recall bias. The caregivers were excluded if they were not able to answer the questions because of having any kind of disability or language problem and/or if they were not willing to participate in the study. Prior to the data collection, we prepared a list of the number of children registered in each relevant center/clinic at both province levels. Therefore, when the interviewers went to the centers, they knew how many participants they might have. Among the 219 caregivers of children with ASD, who were receiving services, in the two provinces, 17 refused to participate in the study (three from Ardabil province and 14 from East-Azerbaijan province) and 202 individuals completed the questionnaire (47 from Ardabil Province and 155 from East-Azerbaijan province). A diagram showing the types of the participants recruited in the study is presented in Fig. 2.

### Measures and instruments

#### Social determinants of health (SDH)

After making some modifications, an instrument extracted from Gottlieb et al.’s work was used to assess the SDH status of children with ASD [26]. The final questionnaire encompasses 18 individual items about the problems in psychosocial areas, which explore how stressful are the main issues related to their income, job safety, insurance and access to healthcare, house-holding, and provision of staples. Likert-scale response options were as follows: 6 = “not at all stressful,” 5 = “a little bit stressful,” 4 = “moderately stressful,” 3 = “very stressful,” 2 = “extremely stressful,” and 1 “issue listed is not applicable to my family”. Total score was normalized in a range from 0 (the lowest SDH) to 100 (the optimum SDH).

#### Quality of services

We considered four domains, including participation in decision making, care coordination, continuity of care, and timeliness, to assess the quality of services; each domain consisted of four questions. The questionnaire was derived from the study of Sun et al. [27] with some modifications in the content and number of questions through a panel of professionals in the fields of health management and/or ASD services. The questions were submitted with dichotomous answer categories (yes/no). The domain of participation in decision making included items in terms of listening to caregivers and encouraging them to ask their questions, involving them in the care process, providing them with adequate information on how to care of their child, and providing them with adequate information on treatment choices. Domain of the care coordination encompassed questions regarding the availability of a coordinator in the center, following-up the next visit, following-up the child’s referral to another center (if applicable), and routine contacts of the center with child’s school, daily-care center, etc. (if applicable). Domain of the continuity of care consisted of items about the receiving services from a specific therapist, receiving services from a specific psychiatrist, adequate visit time, and the appropriate interval between visits. Domain of the timeliness included wasting time for language and occupational therapists and psychiatrist, the time interval between visits, and visit duration. Total score was normalized in a range from 0 (the lowest quality) to 100 (the highest quality).

#### Access to services

We used six domains to assess access to service based on prior researches in a similar field [23, 27]. Domains included problems in service use, problems in referrals, inadequate insurance coverage, cultural and family issues, problems in access to trusted providers, and problems related to waiting time. A dichotomous answer choice (yes/no) was considered as the response format for each question. Domain of the problems in service use included five items: distance from medical/rehabilitation center, failure to make appointment, high cost, lack of awareness regarding the available services, and other explanations. Domain of the problems in referrals encompassed three items in terms of the referral of the child to other centers (if applicable), including difficulty in the referral process, failure of the referrals, and not following up the referrals. Domain of the inadequate insurance coverage contained three items including, failure to receive a specific service or delay in getting that service, failure to receive specialty services, and using services less than what parents felt needed. Domain of the cultural and family issues composed of three questions to explore if family and cultural issues had been a reason that a child had less utilization of services than needed, had left the center before receiving the service, or had not gone to the center for a long time. Domain of the problems in access to trusted providers included four questions which explored if lack of trust in the psychiatry/providers of the service (two separate questions) and not having good communication with them (two separate questions) had made them go to the center less than needed. The last domain, problems of waiting time, contained three items to explore if a long waiting time had been a reason for less utilization of services than needed, leaving the center before receiving the services, or changing the center. Total score was normalized in a range from 0 (the lowest access) to 100 (the highest access).

#### Other demographics and behavioral variables

The background variables included age, gender, mother tongue, father and mother status (alive\divorced\died), father and mother education level (illiterate\primary school\secondary school\high school\diploma\bachelor\master\doctorate or above), father and mother occupations, type of health insurance (Social Security\Health Service\Army\Iranian Health\Oil Company\Other (specify)\Uninsured), and sibling(s) with ASD (yes/no).

### Validity and reliability of the measures

The measure illustrates a good to excellent ranges of internal consistency for the scales: SDH (α =0.924), quality of services (α = 0.704), and access to services (α = 0.792). Questionnaires validity assessed and confirmed using content validity. Construct validity of the measure was assessed and confirmed using confirmatory factor analysis (CFA).

#### Data collection

Two pairs of trained interviewers collected data in each province (four interviewers). After corresponding with the University of Medical Sciences, the Education and Training Organizations, and the Welfare Organization in each province, and getting permission to collect data from related centers, a staff member (a secretary, teacher, or therapist) in each center invited parents of children with ASD to participate in the study providing them with brief information about the research team and the objectives of the study and scheduled the interviews. Interviews lasted 40–50 min on average. After filling and signing the informed consent form, interviewers interviewed with the participants in each center, where their children received services, in a separate room to assure privacy, using a structured questionnaire. Interviewers didn’t interfere in the responses provided by the participants. They just explained the questions to the participants in case of any problem with any questions, regarding the illiterate participants. In the whole process of the interviews, a copy of the questions was provided to each participant and they selected their answers by their own unless they were illiterate. The role of the interviewers was just as a facilitator. At the end of each interview, the interviewers gave a gift worth 200,000 Rial (equal to $2) to each participant. Interviews were conducted in Turkish or Persian language based on the participants’ preferences.

#### Data analysis

Structural equation modeling (SEM) was used to test the fitness of the measurement conceptual model. Goodness of fit indices was used to investigate the fitness of the model. Values smaller than 0.08 for root mean square error of approximation (RMSEA) and values greater than 0.90 for Tucker-Lewis index (TLI) and comparative fit index (CFI) confirmed the fitness of model (33). *P*-values ≤0.05 were considered statistically significant. Missing at random (MAR) assumptions and imputation method was used to address missing data. Data were analyzed using the IBM SPSS Statistics, version 23 (IBM Corp., Armonk, N.Y., USA) and IBM SPSS Amos 24 (IBM Corp., Armonk, N.Y., USA).

## Results

According to the findings, a total of 202 caregivers of children with ASD were recruited in the study, of whom 152 were male (76.7%). The mean age of children was 2 years and 6 months. The fathers of 10 children (5.1%) were dead and the parents of 7 children were divorced. In addition, 7 participants (3.5%) reported not having insurance coverage and 28 participants (14%) stated that they were covered by Iranian Health Insurance for free. (see Table 1).

**Table 1:** Demographic and background characteristics of the children with autism spectrum disorders participated in the study, North-West of Iran, 2019

Variable	No	Percent
Child Gender		
Male	152	76.7
Female	47	23.3
Father status		
Alive	181	91.4
Died	10	5.1
Divorce	7	3.5
Father Education		
Illiterate	12	6.0
High school	108	53.7
Academic	81	40.3
Father Job		
Self-employment	67	35.6
Employee	49	26.1
Manual worker	67	35.6
Unemployed	5	2.7
Mother status		
Alive	189	96.5
Divorce	7	3.5
Mother Education		
Illiterate	12	6.5
High school	108	52.2
Academic	81	41.3
Insurance		
Social Security	107	53.5
Health Service	21	11.5
Military	8	4.5
Iranian Health	28	14.0
Oil Company	26	13.0
Uninsured	7	3.5

Based on the results of the current study, participation in decision making with a mean of 65.61 (36.07) scored highest among the quality dimensions. Also, the overall mean of the quality was 61.23 (30.01), indicating the moderate quality score of services provided to the children. Cultural and family domain got the highest score (84.44 out of 100) and the services use got the worst score (49.73 out of 100) among the access dimensions. The mean of the SDH status of the children was 29.50 out of 100 (see Table 2).

**Table 2:** Social determinants of health, quality, and access scores reported by the caregivers of children with autism spectrum disorders, North-West of Iran, 2019

Variables	Mean*	SD
Quality dimension		
Participation in decision making	65.61	36.07
Care coordination	61.26	37.86
Continuity of care	60.82	40.35
Timeliness	62.72	38.24
Total service quality	61.23	30.01
Access dimension		
Services usage	49.73	32.25
Referral	60.18	23.72
Insurance coverage	51.71	40.86
Cultural and family	84.44	27.12
Access to trusted provider	71.69	32.58
Waiting time	77.72	30.96
Total	65.91	21.89
SDH		
SDH Score	29.50	22.32

* 0–100; 0 = worst condition and 100 = best condition

### Basic model

**Fig. 3:**
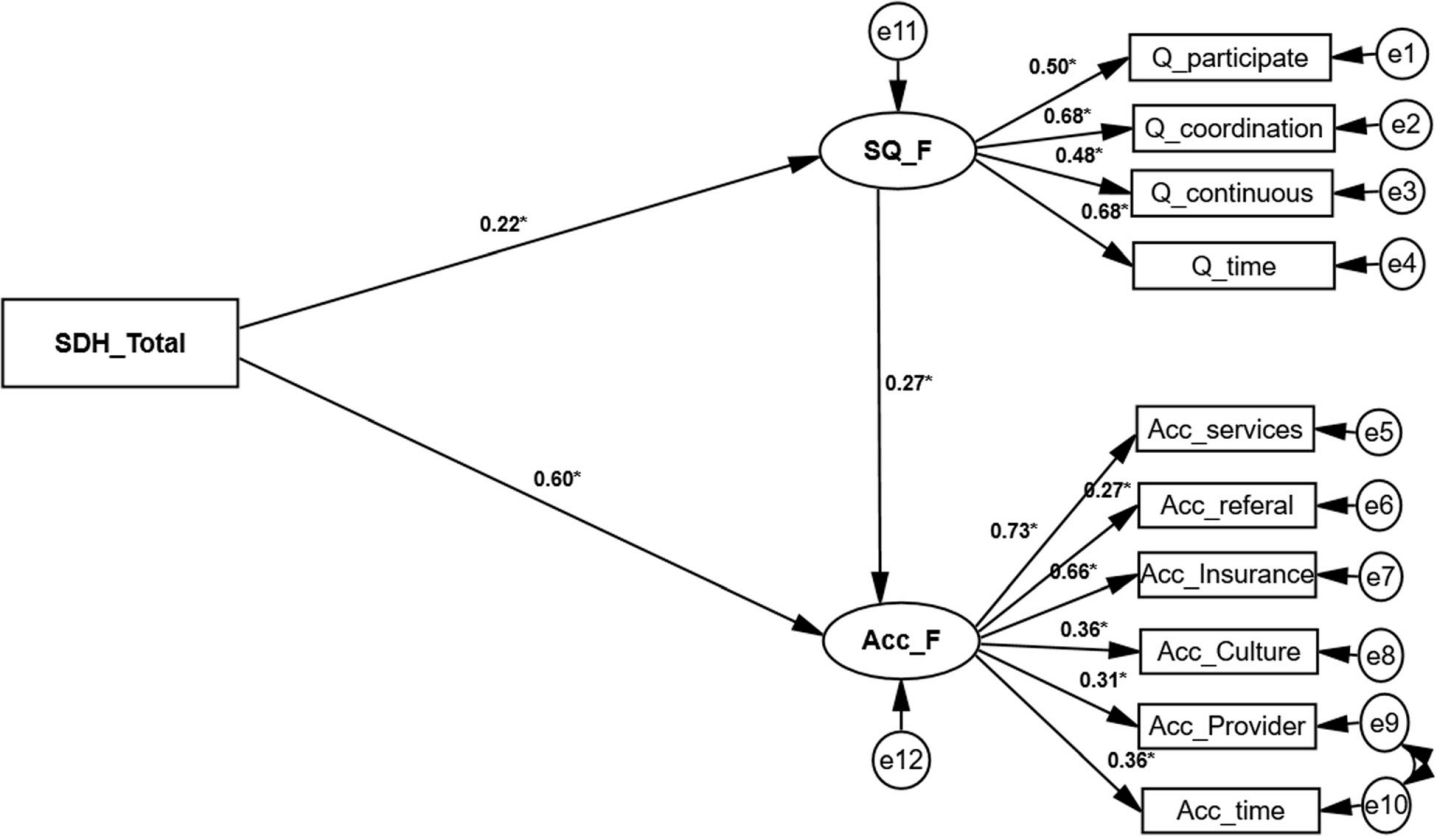
Basic model of relationship between social determinants of health, the quality of and access to services, among children with autism spectrum disorders, North-West of Iran, 2019. Abbreviations: *ASD*, Autism Spectrum Disorder; *SDH*, Social Determinants of Health; *SQ*, Service Quality; *ACC*, Access; *Q_Participate*, Participation Dimensions of the Quality; *Q_Coordination*, Coordination Dimensions of the Quality; *Q_Continuous*, Care Continuity Dimension of the Quality; *Q_Time*, Timeliness Dimension of the Quality; *Ac_Referal*, Referral Dimension of the Access; *Ac_Insurance*, Insurance Dimension of the Access; *Ac_Culture*, Culture Dimension of the Access; *Ac_Provider*, Provider Dimension of the Access; *Ac_Time*, Delayed Time Dimension of the Access; *Ac_Services*, Service Availability Dimension of the Access

The basic model fitted the data well after some modifications; χ2 (41) =78.330, *P* < .001, χ2/df = 1.91 < 5, TLI = 0.86 > 0.8, CFI = 0.9 > 0.8, RMSEA = 0.07 < .08 (90% CI = (0.044 to 0.090). All the associations between the quality and access dimensions and quality (B: 0.464–0.704) and access (B: 0.265–0.726) scales were statistically significant (*P* < 0.001), as was assumed in the conceptual model. Access was also significantly related to service quality (*P* = 0.007). That is, the quality of services was a mediator between SDH and access as well. Finally, in line with the conceptual model, the associations between SDH score with service quality (*P* = 0.014) and access (*P* < 0.001) were positively significant (Fig. 3), also, results are presented in the tabular format in additional file 1.

### Model with covariate

**Fig. 4:**
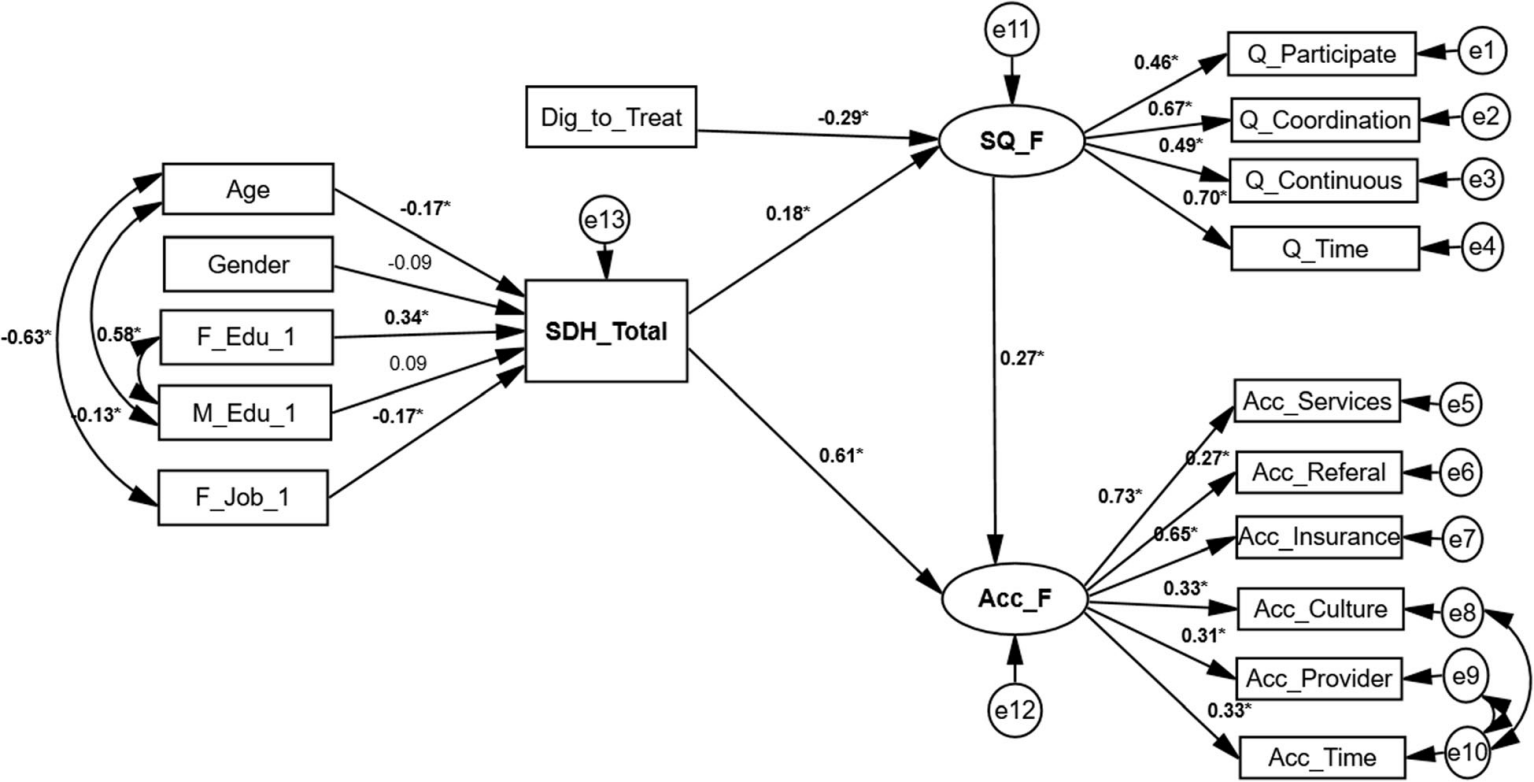
Full model of the associations between social determinants of health, the quality of and access to services among children with autism spectrum disorders by adjusting to covariates, North-West of Iran, 2019. Abbreviations: *SDH*, social determinants of health; *ACC*, access; *Q*, quality; *Q_Participate*, Participation Dimensions of the Quality; *Q_Coordination*, Coordination Dimensions of the Quality; *Q_Continuous*, Care Continuity Dimension of the Quality; *Q_Time*, Timeliness Dimension of the Quality; *Ac_Referal*, Referral Dimension of the Access; *Ac_Insurance*, Insurance Dimension of the Access; *Ac_Culture*, Culture Dimension of the Access; *Ac_Provider*, Provider Dimension of the Access; *Ac_Time*, Delayed Time Dimension of the Access; *Ac_Services*, Service Availability Dimension of the Access; *Edu*, Education, *Dig_to_Treat*, Diagnosis to treatment

The Model with covariate fitted the data well after some modifications; χ2 (116) =202.76, *P* < 0.001, χ2/df = 1.81 < 5, TLI = 0.83 > 0.8, CFI = 0.9 > 0.8, RMSEA = 0.06 < .08 (90% CI = (0.049 to 0.077). In addition, all the associations between the quality and access dimensions, and quality and access scales were statistically significant (*P* < 0.05). The access was also significantly related to service quality (*P* = 0.004). Finally, in the model with covariates, the associations between SDH Score with service quality (*P* = 0.039) and access (*P* < 0.001) by adjusting to child age and gender, father and mother education, father job and diagnose to treat time were positively significant. Finally, positive and significant associations were found between SDH Score and child age, and father education and job (*P* < 0.05) (Fig. 4), also, results are presented in the tabular format in additional file 2.

## Discussion

This is the first study to explore the association between SDH, the quality of services, and access to services in children with ASD in Iran. According to the present study, there is a positive association between SDH and the quality of services and between SDH and access to services in children with ASD. SDH status directly affected access to services and indirectly affected access to services mediated by the quality of services.

According to the results, the lower SDH status the children had the lower quality of services they experienced, which is consistent with the results of a previous study by Magaña in 2012 aimed to explore the racial and ethnic disparities in the quality of care in children with ASD and other developmental disabilities [17]. Magaña showed a significant gap in the quality of services between Black/Latino children and White children as well as between children with ASD and children with other developmental disabilities [17]. A recent study by Zeleke et al. (2019) corroborates our results showing that white children with ASD had more access to a larger variety of services and were more satisfied with their healthcare compared to minority groups [24].

We also found out that children with lower SDH had less access to healthcare and this association was mediated by the quality of services. In line to our results, several studies have highlighted significant disparities in access to and the quality of healthcare among children of racial/ethnic minority backgrounds with ASD or other developmental disabilities [17, 22, 28]. A growing range of evidence highlighted that Latino children diagnosed with ASD have less access to and lower utilization and the quality of healthcare in comparison to their white peers [15, 17, 29]. Durkin et al. (2015) showed a major disparity in access to a comprehensive variety of services for children with ASD, especially in low-resources settings [30]. Since most services needed by children with ASD for their specific condition is delivered by private organization affiliated to the Welfare Organization, it is not surprising to see such inequality in access to services in Iran. Although the Welfare Organization provide a subside for the families to use those services, the eligibility criteria are not clear. It is really challenging for families, who have major difficulties in provision of their basic needs, to find and access high-quality, appropriate, and affordable services for their child with ASD. There is only one public autism center in the north-west of Iran. Nevertheless, health insurance does not have coverage for the services and the parents must pay most of the fees of their pocket.

In our study, children with ASD experienced lower access to services in all domains including problems in access to services, problems in referrals, lack of insurance coverage, cultural and family issues, problems in access to a trusted provider, and long waiting time. On the other hand, quality of care was considered as a determinant to access to care. This is somewhat in line with the study of Vohra et al. (2014) in the US, which showed that children with ASD had lower access to services than children without ASD, due to difficulties in using services, difficulties in getting referrals, and inadequate insurance coverage. On the other hand, Vohra showed that children with ASD experienced lower quality of services than those without ASD, in terms of lack of shared decision making and lack of coordination [23]. In our study, although participants reported low access to services due to inadequate insurance coverage, we didn’t adjust the main variables with the type of health insurance, because, in Iran, except Military and Oil Company insurance, other insurance do not have coverage for ASD services. In fact, there is no specific strategy in Iran to guarantee the access of people with disabilities to healthcare services and adequate insurance coverage. Therefore, it is essential for policymakers in the health system to find a solution to guarantee the full coverage of these vulnerable groups [31]. Barriers to accessing the services needed by children with ASD may have further negative consequences. The significance of the problem in access to services is highlighted by Lindly et al. (2019), which indicated the association between problems in access to healthcare and greater emergency department use among children with ASD [32].

Although our sample size was not very big, it was large enough to test model fitness. Our study is certainly generalizable to the whole country (Iran), because, we have recruited all eligible participants in two representative provinces of Iran. On the other hand, as mentioned earlier, children in every part of Iran receive services in a similar structure. Additionally, our results and methodology could be applied by other researchers in other countries to explore socioeconomic disparities in access to and the quality of services in children with not only ASD but other developmental disorders as well.

### Limitations

We believe that there are some limitations in our study. First, we used self-reported questionnaire to assess SDH status of the families. However, we collected data by third party trained and skilled interviewers who had explained each question critically and carefully for interviewees in case of any ambiguity and encouraged them to select the exact answer choice without interfering in their choice. In fact, contribution of the interviewers was to support the interviewee. On the other hand, the interviewers didn’t have any role in the diagnosis or treatment of children with ASD nor did they have any kind of relationship with centers provided services for the children. Despite all mentioned consideration to avoid information bias, although we couldn’t exclude a possible information bias, if there would have been any, we expect it to be small. Second, data on people that had not have contact with the health system were not included in the study and we had our access evaluation among those subjects with different service uses. As we mentioned earlier in the method section, the participants needed to have a minimum use of services to be able to evaluate their access to high-quality services. After reviewing the autism registry data in East-Azerbaijan province, we realized that a total of 550 children with ASD were registered in the registry system. Among them, 130 children were higher than 16-year-old and 300 cases had been entered in the data-base retrospectively from the Welfare Organization documents, for whom there were not diagnosis documents verified by a psychiatrist. We found out that around 200 children with ASD at the age of 2–16 years did not receive services from any relevant centers/clinics in East-Azerbaijan province. We also received some unofficial information on the total number of children with ASD in Ardabil province showing that approximately 150 children were identified with ASD. Nonetheless, we just could access the information of children who were receiving services from the relevant facilities. We have informally been informed on children without any access to the services not being included in the study so that we cannot evaluate possible selection bias. Finally, this study was conducted in two provinces of Iran; nevertheless, we collected data approximately from all children with ASD who were receiving care from related educational and rehabilitation centers. East-Azerbaijan province is the sixth most populated province among 31 provinces of Iran, with 3.8 million population of which 28% live in rural areas similar to the national proportion (26% of the population of Iran live in rural areas). Ardabil province, on the other hand, with 1.8 million population ranked 23 among 31 provinces. These two provinces altogether have made our target population good representatives of Iran population. Also, services for children with ASD are similar in all regions in our country.

## Conclusion

In conclusion, we found that there are socioeconomic disparities in the quality of and access to services in children with ASD, who use ASD services, in the North-West of Iran. This gap is partially because these children’s main services, which are rehabilitation therapies, are not covered by health insurance and are not provided by primary health centers. Most of the services are provided by private organizations affiliated to the Welfare Organization.

Policy-makers should initiate some measures to increase the coverage of the main services needed by these children, including speech and occupation therapies, through increasing the number of facilities that provide ASD services. Additionally, the Ministry of Health or the Welfare Organization should take some actions to create some level of integration among the facilities. As is mentioned in the limitation of the study as well, many children do not receive services from any of the facilities and many have experienced a long waiting time to be admitted by the centers to initiate their treatment due to the low capacity of the facilities, while some children receive services from more than one facility at the same time. If facilities work in close collaboration with each other, many problems in terms of access and quality could be solved. Recently, the Higher Health Insurance Council has added ASD to the list of the Special Diseases, which means that these children could receive financial subsidies for their services. Therefore, we believe that increasing the number and capacity of facilities could result in significant improvement in equal access to high-quality services.

We recommend health/medical centers, where children are diagnosed with ASD, conducting SDH screening and providing families with low-SDH status with information on the quality of and access to services in order to assist them to overcome barriers in access to high-quality services needed for their children with ASD. Additionally, more researches are needed to assess the utilization of and access to services among all children diagnosed with ASD and to identify the whole range of barriers especially among those with no access to services. Our instruments could be adopted by other researchers in both developing and developed countries to assess the socioeconomic disparities in access to and quality of services in the population of children with ASD.

## Supplementary Information

**Additional file 1.** Relationship between social determinants of health and the quality of and access to services, North-West of Iran, 2019. Results of the basic model are presented in the tabular format.

**Additional file 2.** Relationship between social determinants of health, the quality of and access to services among children with ASD by adjusting to covariates, North-West of Iran, 2019. Results of the Model with covariate are presented in the tabular format.

## Data Availability

The datasets used and analyzed during the current study are available from the corresponding author on reasonable request.

## Abbreviations

ASD: Autism Spectrum Disorders
WHO: World Health Organization
LMICs: Low-and middle-income countries
US: Unite States
SDH: Social Determinants of Health
ABBILAR: Azeri Blue Buddies: Interdisciplinary Longitudinal Autism Researches
CFA: Confirmatory Factor Analysis
SEM: Structural Equation Modeling
RMSEA: Root Mean Square Error of Approximation
TLI: Tucker-Lewis index
CFI: Comparative Fit Index
MAR: Missing at Random
