# Supplementary material for: Disparities in the quality of and access to services in children with autism spectrum disorders: a structural equation modeling
Source: Arch Public Health. 2021 Apr 26;79:58. doi: 10.1186/s13690-021-00577-5 (PMC8074455; doi:10.1186/s13690-021-00577-5)
Supplement: Supplementary file 1 — Additional file 1. Relationship between social determinants of health and the quality of and access to services, North-West of Iran, 2019. Results of the basic model are presented in the tabular format. [file 13690_2021_577_MOESM1_ESM.docx]

Additional file 1

Table 1. Relationship between social determinants of health and the quality of and access to services among children with autism spectrum disorders, North-West of Iran, 2019

| **Variables** | | | **Estimate** | **S.E.** | **Standardized Estimate** | **C.R.** | **P** |
| --- | --- | --- | --- | --- | --- | --- | --- |
| SQ_F | ← | SDH_Total | .169 | .069 | .227 | 2.452 | .014 |
| ACC_F | ← | SDH_Total | .628 | .085 | .602 | 7.383 | <0.001 |
| ACC_F | ← | SQ_F | .376 | .140 | .269 | 2.679 | .007 |
| Q_participate | ← | SQ_F | 1.000 |  | .420 |  |  |
| Q_coordination | ← | SQ_F | 1.529 | .335 | .672 | 4.563 | <0.001 |
| Q_continuous | ← | SQ_F | 1.157 | .286 | .477 | 4.049 | <0.001 |
| Q_time | ← | SQ_F | 1.606 | .351 | .699 | 4.571 | <0.001 |
| Ac_referal | ← | ACC_F | .278 | .084 | .273 | 3.326 | <0.001 |
| Ac_Insurance | ← | ACC_F | 1.154 | .160 | .658 | 7.231 | <0.001 |
| Ac_Culture | ← | ACC_F | .414 | .097 | .355 | 4.281 | <0.001 |
| Ac_Provider | ← | ACC_F | .437 | .116 | .312 | 3.771 | <0.001 |
| Ac_time | ← | ACC_F | .470 | .111 | .354 | 4.253 | <0.001 |
| Ac_services | ← | ACC_F | 1.000 |  | .722 |  |  |

**Abbreviations**: ASD, Autism Spectrum Disorder; SDH, Social Determinants of Health; SQ, Service Quality; ACC, Access; Q_Participate, Participation Dimensions of the Quality; Q_Coordination, Coordination Dimensions of the Quality; Q_Continuous, Care Continuity Dimension of the Quality; Q_Time, Timeliness Dimension of the Quality; Ac_Referal, Referral Dimension of the Access; Ac_Insurance, Insurance Dimension of the Access; Ac_Culture, Culture Dimension of the Access; Ac_Provider, Provider Dimension of the Access; Ac_Time, Delayed Time Dimension of the Access; Ac_Services, Service Availability Dimension of the Access.
