# Supplementary material for: Disparities in the quality of and access to services in children with autism spectrum disorders: a structural equation modeling
Source: Arch Public Health. 2021 Apr 26;79:58. doi: 10.1186/s13690-021-00577-5 (PMC8074455; doi:10.1186/s13690-021-00577-5)
Supplement: Supplementary file 2 — Additional file 2. Relationship between social determinants of health, the quality of and access to services among children with ASD by adjusting to covariates, North-West of Iran, 2019. Results of the Model with covariate are presented in the tabular format. [file 13690_2021_577_MOESM2_ESM.docx]

Additional file 2

Table 2. Relationship between social determinants of health, the quality of and access to services among children with ASD by adjusting to covariates, North-West of Iran, 2019

| **Variables** | | | **Estimate** | **S.E.** | **Standardized Estimate** | **C.R.** | **P** |
| --- | --- | --- | --- | --- | --- | --- | --- |
| SDH_Total | ← | Gender | -4.981 | 3.329 | -1.496 | -.095 | .135 |
| SDH_Total | ← | Age | -.069 | .033 | -2.097 | -.174 | .036 |
| SDH_Total | ← | F_Edu_1 | 12.919 | 2.975 | 4.342 | .340 | <0.001 |
| SDH_Total | ← | M_Edu_1 | 3.528 | 3.004 | 1.174 | .093 | .240 |
| SDH_Total | ← | F_Job_1 | -4.271 | 2.027 | -2.107 | -.173 | .035 |
| SQ_F | ← | SDH_Total | .132 | .064 | 2.063 | .176 | .039 |
| SQ_F | ← | Dig_to_Treat | -5.287 | 1.679 | -3.148 | -.290 | .002 |
| ACC_F | ← | SDH_Total | .636 | .084 | 7.538 | .610 | <0.001 |
| ACC_F | ← | SQ_F | .383 | .134 | 2.852 | .275 | .004 |
| Q_Participate | ← | SQ_F | 1.000 |  |  | .464 |  |
| Q_Coordination | ← | SQ_F | 1.514 | .299 | 5.061 | .671 | <0.001 |
| Q_Continuous | ← | SQ_F | 1.169 | .265 | 4.413 | .485 | <0.001 |
| Q_Time | ← | SQ_F | 1.604 | .315 | 5.094 | .704 | <0.001 |
| Ac_Referal | ← | ACC_F | .270 | .084 | 3.224 | .265 | .001 |
| Ac_Insurance | ← | ACC_F | 1.145 | .160 | 7.139 | .655 | <0.001 |
| Ac_Culture | ← | ACC_F | .390 | .097 | 4.025 | .335 | <0.001 |
| Ac_Provider | ← | ACC_F | .433 | .116 | 3.738 | .310 | <0.001 |
| Ac_Time | ← | ACC_F | .439 | .110 | 3.977 | .332 | <0.001 |
| Ac_Services | ← | ACC_F | 1.000 |  |  | .726 |  |

**Abbreviations**: ASD, Autism Spectrum Disorder; SDH, Social Determinants of Health; SQ, Service Quality; ACC, Access; Q_Participate, Participation Dimensions of the Quality; Q_Coordination, Coordination Dimensions of the Quality; Q_Continuous, Care Continuity Dimension of the Quality; Q_Time, Timeliness Dimension of the Quality; Ac_Referal, Referral Dimension of the Access; Ac_Insurance, Insurance Dimension of the Access; Ac_Culture, Culture Dimension of the Access; Ac_Provider, Provider Dimension of the Access; Ac_Time, Delayed Time Dimension of the Access; Ac_Services, Service Availability Dimension of the Access; Edu, Education, Dig_to_Treat, Diagnosis to treatment.
